# Age-Associated Behavioral Alterations in Laboratory-Housed *Octodon degus*

**DOI:** 10.3390/biology15161352

**Published:** 2026-08-10

**Authors:** Huiru Bai, Yiran Liu, Andrei Seluanov, Vera Gorbunova

**Affiliations:** Department of Biology, University of Rochester, Rochester, NY 14627, USA; hbai6@ur.rochester.edu (H.B.); yliu311@u.rochester.edu (Y.L.)

**Keywords:** *Octodon degus*, aging, locomotor activity, open field, social novelty, three-chamber test, ethological scoring, laboratory housing, neurodegeneration model

## Abstract

*Octodon degus* are long-lived, diurnal, and highly social rodents increasingly used in studies of aging and neurodegeneration. However, behavioral profiles of aged laboratory-housed degus remain incompletely characterized, limiting the interpretation of aging-associated functional and molecular phenotypes in this species. Here, we evaluated age-associated changes in locomotor activity, open-field exploration, social novelty behavior, and manually scored ethological responses in young and old degus maintained under long-term laboratory housing conditions. Automated behavioral tracking was performed during open-field testing and three-chamber social behavior testing. During open-field testing, old degus showed increased locomotor activity compared with young animals, including greater total distance moved, higher mean velocity, increased moving frequency, and longer cumulative movement duration. Old degus also showed increased center-zone duration and reduced thigmotaxis score. Manual ethological scoring revealed increased rearing and fecal boli in old animals during open-field exposure. In the three-chamber social behavior assay, young degus showed higher investigation frequency toward the novel intruder than toward the familiar cagemate, whereas old degus showed a lower social novelty discrimination index compared with young animals. Exploratory sex-stratified analyses identified selective differences in center–border exploration in the young cohort: young males spent more time in the center zone and showed lower thigmotaxis than young females, whereas no significant sex-associated differences were detected in the other open-field or social novelty measures examined. Together, these findings indicate that aging in laboratory-housed degus is associated with a mixed behavioral profile involving increased stress-related ethological responses and reduced social novelty discrimination, affecting behavioral domains relevant to dementia and neurodegenerative aging. This behavioral framework provides a practical reference for future studies examining behavioral heterogeneity and molecular or neuropathological correlates of brain aging in degus.

## Simple Summary

*Octodon degus* are long-lived, highly social rodents that are increasingly used to study aging and age-related neurodegenerative disorders. However, interpreting behavioral changes in aged degus requires a clear understanding of how aging affects activity, exploration, social behavior, and stress-related responses under laboratory housing conditions. In this study, we compared young and old degus using open-field testing, three-chamber social behavior testing, automated video tracking, and manual scoring of selected behaviors. Aged degus did not show a simple reduction in behavioral activity. Instead, they showed increased movement during behavioral testing, increased rearing behavior, greater exploration of the center of the open-field arena, and increased fecal output during open-field exposure. These findings suggest that aged degus show increased exploratory activity together with altered stress-related responses in a novel environment. Aged degus also showed reduced social novelty discrimination, a behavioral change relevant to dementia-associated social and cognitive domains, although molecular and neuropathological analyses are needed to determine its relationship to AD-like pathology. Together, these results define a behavioral profile of aged laboratory-housed degus and provide a practical reference for future studies using this species to investigate aging, social behavior, and neurodegeneration-related phenotypes.

## 1. Introduction

The degu (*Octodon degus*), a long-lived, diurnal, and highly social South American rodent belonging to the family Octodontidae, is increasingly used to investigate natural aging and age-associated neurological changes [1,2,3]. Some aged degus have been reported to exhibit behavioral and cognitive impairments together with AD-like molecular and neuropathological changes, including amyloid-β accumulation, tau-related alterations, synaptic dysfunction, and neuroinflammation [4,5,6,7]. However, these features have not been consistently observed across individuals or captive colonies [6,8,9]. This variability complicates interpretation of the model and highlights the need for rigorous behavioral phenotyping under well-defined laboratory conditions.

Previous studies have identified age-sensitive behavioral phenotypes in degus, although their direction and magnitude vary among cohorts and experimental designs. Popović et al. reported reduced open-field ambulation and rearing in 40-month-old females compared with 23-month-old females, together with time-of-day-dependent differences in anxiety-related measures [10]. Ardiles et al. linked impaired spatial and object-recognition memory to synaptic dysfunction and AD-like molecular features [4]. Rivera et al. examined social, object, and location recognition and Barnes maze performance in females aged 12–75 months. They identified age-dependent changes in short- and long-term memory, with the clearest impairment in short-term recognition, but found no overall age-related difference in open-field locomotion [11]. Similarly, Oliva et al. found preserved locomotion and sociability but reduced social novelty preference and impaired long-term spatial memory in aged vehicle-treated females in a long-term pharmacological study [12]. Burrowing assays revealed additional heterogeneity: impaired burrowing identified subsets of aged animals with more prominent neuroinflammatory and AD-like neuropathological features [5,6].

Environmental and experimental factors may further shape these phenotypes. Cavieres et al. used open-field and three-chamber assays to examine the behavioral consequences of prolonged social isolation [13]. In related work, Oliva et al. reported sex-dependent changes in neurodegeneration-related markers associated with long-term social isolation [14]. More recently, Rivera et al. evaluated diet-associated differences in open-field, three-chamber, and recognition measures in four-year-old males [15]. Differences in sex composition, age distribution, colony origin, diet, housing, and experimental exposure may therefore contribute to variation among studies.

Despite these advances, most studies of natural behavioral aging have examined single-sex cohorts, whereas other multidomain studies have focused on pharmacological intervention, social isolation, diet, or behaviorally stratified subgroups. It therefore remains unclear whether reduced social novelty investigation in aged degus can be explained by a generalized reduction in activity or instead occurs despite preserved or increased locomotor and exploratory activity. Resolving this question requires concurrent assessment of locomotor, exploratory, and social behaviors within the same animals.

Here, we combined automated open-field and three-chamber tracking with blinded manual scoring of rearing, grooming, jumping, and fecal boli production in young adult and aged laboratory-housed degus from a long-term breeding colony. Both males and females were included, allowing exploratory assessment of within-age sex differences. Aged degus did not exhibit generalized behavioral suppression. Instead, they showed greater locomotor and exploratory activity, increased rearing, and greater fecal boli production. Together, these changes are consistent with altered arousal and stress responsiveness to a novel environment. Despite this increased behavioral activation, aged animals showed reduced social novelty discrimination. These findings define a domain-specific profile of behavioral aging and provide a framework for future studies linking behavioral heterogeneity to sensory, molecular, endocrine, and neuropathological variation.

## 2. Materials and Methods

### 2.1. Animals

**Figure 1:**
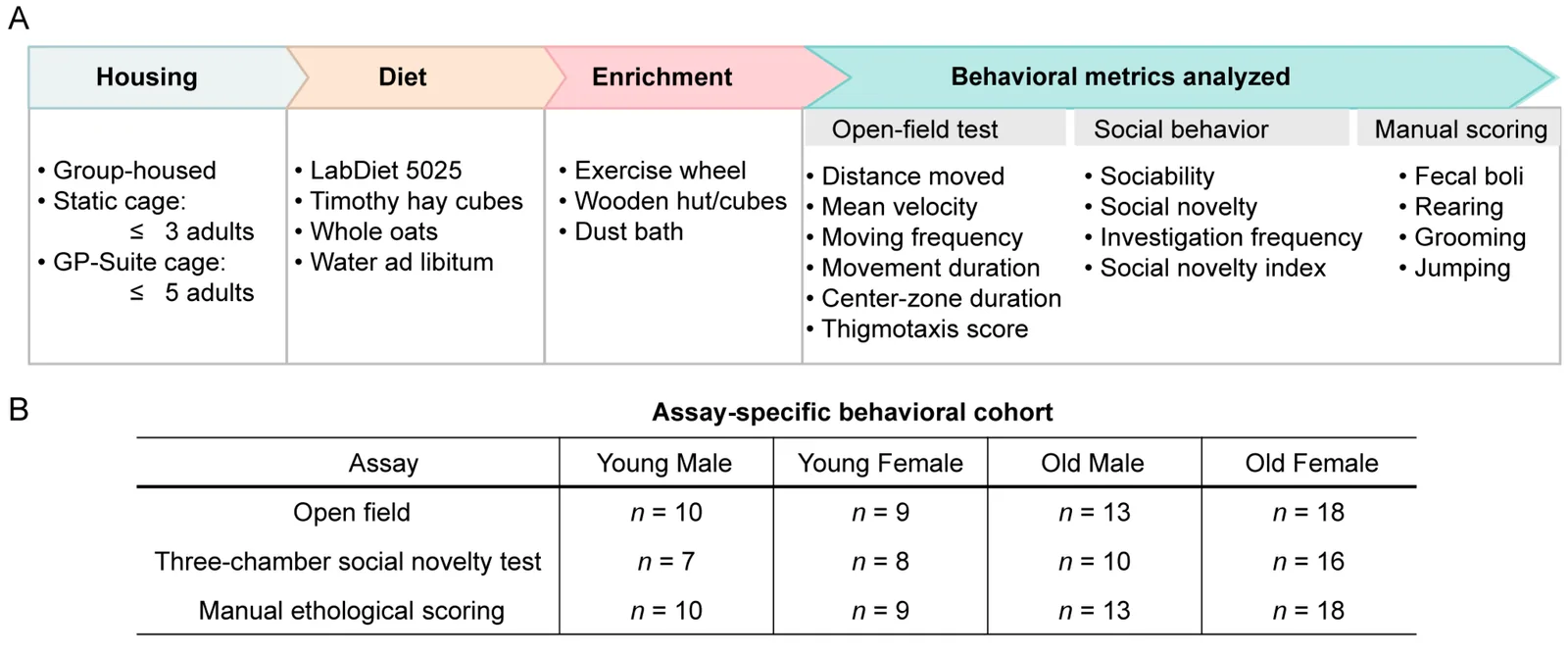
Study design and behavioral analysis overview. (**A**) Schematic overview of laboratory housing and husbandry conditions, behavioral workflow, and analyzed behavioral metrics. Open-field analyses included locomotor tracking and center-border exploration measures during a 5 min recording window. Three-chamber social behavior testing included three sequential 10 min phases: habituation, sociability, and social novelty. Sniffing-zone entry frequency and social novelty discrimination index were quantified during the social novelty phase. Manual ethological scoring was performed over a 5 min open-field exposure and included fecal boli, rearing, grooming, and jumping. (**B**) Assay-specific cohort structure for open-field testing, three-chamber social behavior testing, and manual ethological scoring. Young adult animals were 0.3–1 years of age, and old animals were 4–7 years of age. Values indicate the number of animals included in each assay or analysis.

*Octodon degus* were maintained in the laboratory colony at the University of Rochester under a 12:12-h light–dark cycle, with lights on at 06:00 and off at 18:00. Animals were assigned to a young cohort (0.3–1 years of age) or an old cohort (4–7 years of age), and both males and females were included. Before behavioral testing, animals underwent routine veterinary health assessment. Prespecified exclusion criteria included overt illness or injury, impaired mobility, clinically significant skin or coat abnormalities, and a loss of ≥20% relative to the animal’s most recent stable body weight. No animal tested in this study met any of these exclusion criteria. Assay-specific sample sizes are provided in Figure 1B. Open-field locomotor tracking and manual ethological scoring were performed in 19 young and 31 aged degus. The three-chamber assay included 15 young and 26 aged degus. Because a familiar cagemate was required for the social-novelty paradigm, this assay was restricted to animals with an available and eligible cagemate; animals without a suitable cagemate were not tested in the three-chamber assay. Thus, the smaller sample size in the three-chamber assay reflected cagemate availability rather than exclusion based on behavioral performance.

All animal procedures were performed in accordance with institutional guidelines and were approved by the University Committee on Animal Resources at the University of Rochester under protocol UCAR-2009-054R.

### 2.2. Housing and Husbandry

Degus were maintained under long-term laboratory housing conditions with group housing, standardized diet, bedding, and environmental enrichment [16,17]. Two primary cage systems were used for adult colony housing. The original cage system consisted of Tecniplast Static Cage 2000P units with 320 in^2^ of floor space, including a cage base, wire top, and food hopper (Tecniplast, Tecniplast Static Cage 320 in^2^-2000P, product code 2000P001, West Chester, PA, USA). These cages housed up to three adult degus and could accommodate an exercise wheel. A larger cage system consisted of Tecniplast GP-Suite guinea pig cages, each providing 618 in^2^ of floor space and measuring approximately 31.875 × 28.000 × 9.875 inches (Tecniplast, GP-SUITE—Rack for Guinea Pigs, West Chester, PA, USA). These cages housed up to five adult degus and allowed placement of both an exercise wheel and a shelter.

Biofresh Comfort Bedding (ScottPharma Solutions, L0110 Biofresh Comfort Bedding Natural 60 L, Marlborough, MA, USA) was used as bedding, and each cage was provided with an Enviropak (ScottPharma Solutions, EnviroPak, Marlborough, MA, USA). Environmental enrichment included Exotic Nutrition exercise wheels (Exotic Nutrition Pet Supply, Treadmill Wheel, Newport News, VA, USA), wooden huts or wooden cubes for chewing, stainless steel toys, and weekly dust baths. Dust baths were provided using Blue Beauty Dust (Lixit Corporation, Napa, CA, USA) placed in a stainless-steel bowl, typically the day before cage changes.

Degus were fed LabDiet 5025 Guinea Pig Diet (LabDiet, 5025—Guinea Pig Diet, Gray Summit, MO, USA) daily using cage food hoppers. Plain tap water was provided ad libitum in water bottles. Animals also had continuous access to Timothy hay cubes (ScottPharma Solutions, Timothy hay cubes, Marlborough, MA, USA) and received small amounts of whole oats sprinkled into the bedding several times per week, particularly during cage changes, to encourage foraging behavior. Animals were monitored as part of routine colony husbandry and veterinary care procedures.

### 2.3. Study Design, Cohort Structure, and Behavioral Workflow

To establish a baseline behavioral framework for aged laboratory-housed *Octodon degus*, we compared young adult and old animals across locomotor, exploratory, social, and manually scored ethological behavioral domains. Animals were maintained under long-term laboratory housing conditions with group housing, standardized diet, and environmental enrichment. The behavioral workflow consisted of open-field testing, three-chamber social behavior testing, automated video tracking, and manual ethological scoring (Figure 1A). Open-field tracking was used to quantify locomotor activity and center-border exploration measures, including total distance moved, mean velocity, moving frequency, cumulative movement duration, center-zone duration, and area-corrected thigmotaxis score. Three-chamber analyses were used to assess baseline zone preference, sociability, social novelty behavior, sniffing-zone entry frequency, and social novelty discrimination index. Manual ethological scoring was used to quantify fecal boli, rearing, grooming, and jumping during open-field exposure.

Young adult animals were 0.3–1 years of age, and old animals were 4–7 years of age. Sample sizes were reported separately for each assay and analysis. Open-field locomotor tracking and manual ethological scoring included 19 young and 31 old degus, whereas the three-chamber social behavior assay included 15 young and 26 old degus because this paradigm required an available and eligible familiar cagemate. Animals with zero cumulative duration in both relevant sniffing zones were excluded only from the corresponding discrimination-index analysis because the index was undefined.

### 2.4. Open-Field Behavioral Testing

Open-field behavior was assessed in a square arena measuring 100 cm × 100 cm with 40 cm high gray walls and a white floor. This color configuration provided sufficient contrast between the animals and the arena background for automated video tracking. All animals were naïve to the open-field arena and were tested only once. Each animal was placed individually in the arena and allowed to explore freely for 5 min. Testing was completed over two consecutive days within the same daily time window (13:30–18:00), with males tested on the first day and females on the following day. Both age groups were represented on each testing day. The testing room was kept quiet, and access was restricted during video-recording. One experimenter handled and placed the animal in the arena, whereas a second experimenter operated the video-recording system. Behavioral videos were recorded from above and subsequently analyzed using EthoVision XT 16 software (Noldus Information Technology, Wageningen, the Netherlands). The arena was cleaned with 70% ethanol between animals and allowed to dry before the next trial.

For center-border analysis, the arena was divided into a central square zone measuring 50 cm × 50 cm and a surrounding border zone. The center zone represented 25% of the total arena area, whereas the border zone represented the remaining 75%.

### 2.5. Three-Chamber Social Behavior Testing

Social behavior was assessed using a three-chamber apparatus measuring 120 cm in total length and consisting of three equally sized 40 cm long chambers connected by openings that permitted unrestricted movement between adjacent chambers. Only animals with an available and eligible familiar cagemate were included in this assay. Each test subject underwent the complete three-chamber assay only once. The assay comprised three consecutive 10 min phases—habituation, sociability, and social novelty—for a total duration of 30 min per subject.

During the habituation phase, the test subject was allowed to explore the empty apparatus freely. During the sociability phase, the subject’s current cagemate was placed inside a wire enclosure in one side chamber, while an identical empty wire enclosure was placed in the opposite side chamber. During the social-novelty phase, a novel intruder was placed inside the previously empty enclosure, while the familiar cagemate remained in the opposite enclosure. The intruder was obtained from a different cage, had not previously interacted with the test subject, and was matched to the subject by sex, approximate age and body weight. The side assigned to the familiar cagemate was counterbalanced across subjects when possible; the novel intruder was subsequently introduced into the enclosure on the opposite side.

Testing was conducted in a quiet behavioral-testing room between 09:00 and 18:00 over a single 5-day period. Old males were tested on day 1, young males on day 2, old females on days 3 and 4, and young females on day 5. Access to the room was restricted during video-recording. One experimenter handled and placed the test animals and social stimuli, whereas a second experimenter operated the video-recording system. Videos were recorded from above and subsequently analyzed using EthoVision XT 16 software (Noldus Information Technology, Wageningen, The Netherlands). The apparatus and wire enclosures were cleaned with 70% ethanol between subjects and allowed to dry before the next assay.

### 2.6. Manual Ethological Scoring

To complement automated locomotor tracking, selected ethological behaviors were manually scored from open-field recordings over the same 5 min observation period. The scored behaviors included fecal boli, rearing events, grooming events, and jumping events. Fecal boli were quantified as the total number of fecal pellets produced during the scoring window. Rearing was scored when the animal raised its forelimbs from the floor in a vertical exploratory posture. Grooming was scored as visible self-grooming behavior. Jumping was scored when the animal lifted all four paws from the floor in a vertical jumping movement. Manual scoring was performed from recorded videos using the same predefined behavioral criteria for all animals, and scored values were analyzed as event counts over the 5 min open-field exposure.

### 2.7. Behavioral Tracking and Data Processing

Behavioral videos were analyzed using EthoVision XT 16. For open-field locomotor tracking, the center-point position of each animal was tracked throughout the recording. For the three-chamber assay, center-point tracking was used to quantify chamber-based locomotor measures, whereas nose-point tracking was used to quantify enclosure-associated sniffing-zone measures. All completed open-field and three-chamber recordings passed visual quality inspection and were suitable for the planned automated tracking and manual scoring analyses.

For open-field analysis, automated tracking metrics included total distance moved, mean velocity, moving frequency, cumulative movement duration, center-zone cumulative duration, and area-corrected thigmotaxis score. Moving frequency was defined as the number of movement bouts detected during the recording window, and cumulative movement duration was defined as the total time classified as moving by the tracking software. Center-zone cumulative duration was quantified from center-point tracking data. The area-corrected thigmotaxis score was calculated as the percentage of time spent in the border zone minus the expected border-zone area proportion of 75%. Thus, positive values indicate greater-than-expected border-zone occupancy, whereas values closer to zero indicate border-zone occupancy closer to the area-based expectation.

For chamber-based locomotor analysis, total distance moved and mean velocity were calculated over the 10 min tracking window. These locomotor metrics were analyzed within each assay. Raw locomotor values were not directly compared across assays with different recording durations or apparatus configurations.

For the three-chamber assay, enclosure-associated sniffing zones were defined as 5 cm zones surrounding each wire enclosure. Social investigation was quantified using the nose point of the test animal. Sniffing-zone entry frequency was defined as the number of nose-point entries into each sniffing zone, and cumulative sniffing-zone duration was defined as the total time the nose point remained within each sniffing zone. These measures were quantified for the relevant zones in each phase.

During the habituation phase, occupancy of the two empty enclosure-associated sniffing zones was examined to assess baseline zone bias before the introduction of social stimuli. During the sociability phase, the sociability discrimination index was calculated as (cagemate − empty enclosure)/(cagemate + empty enclosure), using cumulative sniffing-zone duration. During the social novelty phase, the social novelty discrimination index was calculated as (novel intruder − familiar cagemate)/(novel intruder + familiar cagemate), using cumulative sniffing-zone duration. Animals with zero cumulative duration in both zones used for a given discrimination index had an undefined index, and were excluded from the corresponding index-based analysis only.

### 2.8. Statistical Analysis

Statistical analyses were performed using GraphPad Prism 10. For descriptive purposes, data are presented as mean ± standard deviation (SD), with individual animals shown as individual data points. The individual animal was used as the experimental unit.

Comparisons between young and old animals were performed using two-tailed Mann–Whitney U tests for open-field locomotor and center–border measures, chamber-based locomotor measures, the social novelty discrimination index, and manually scored ethological behaviors. A rank-based nonparametric approach was applied consistently without preliminary dataset-specific normality testing. This approach was selected because the behavioral outcomes encompassed count variables, duration measures that included zero values and skewed distributions, and bounded index data obtained from modest-sized cohorts; accordingly, Gaussian distributions were not assumed.

During the social novelty phase, investigation frequency and cumulative sniffing-zone duration directed toward the familiar cagemate and novel intruder were compared within animals using two-tailed Wilcoxon matched-pairs signed-rank tests, performed separately for each age group. Animals with zero cumulative duration in both sniffing zones were excluded from discrimination-index analyses because the index could not be calculated. The proportions of animals with a social novelty discrimination index ≤ 0 were compared between age groups using a two-tailed Fisher’s exact test. Sex-stratified analyses were exploratory and were conducted separately within each age group using two-tailed Mann–Whitney U tests. All tests were two-sided, and nominal *p* < 0.05 was considered statistically significant.

Sample sizes were determined by the availability of age-eligible animals and the number of completed behavioral recordings for each assay during the study period. All completed open-field and three-chamber recordings passed visual quality inspection, and all eligible animals with completed recordings were included in the corresponding analyses. No formal a priori sample size or power calculation was performed. Age-group assignment was based on chronological age and therefore could not be randomized. Behavioral testing was conducted by two investigators as described above. Manual ethological scoring was performed using de-identified recordings by an investigator who was blinded to animal identity, age group, and sex.

## 3. Results

### 3.1. Aged Degus Show Increased Locomotor Activity and Reduced Thigmotaxis in the Open-Field Arena

**Figure 2:**
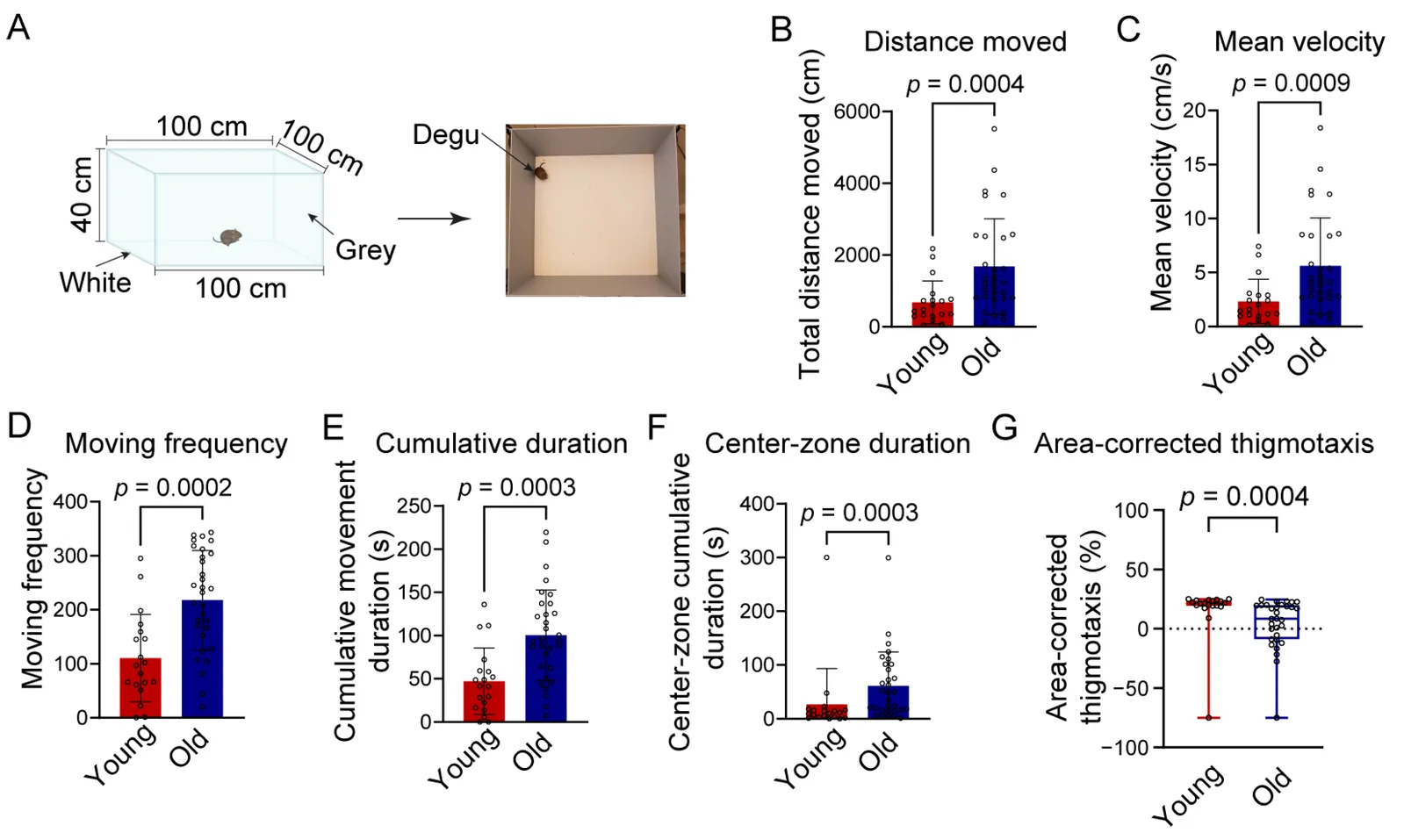
Open-field locomotor activity and center-border exploration in young and old degus. (**A**) Schematic and representative image of the open-field arena used for behavioral testing. (**B**–**E**) Quantification of automated open-field locomotor metrics, including total distance moved (**B**), mean velocity **(C**), moving frequency (**D**), and cumulative movement duration (**E**). (**F**,**G**) Quantification of center-zone cumulative duration (**F**) and area-corrected thigmotaxis score (**G**) as center–border exploration measures. The area-corrected thigmotaxis score was calculated as the percentage of time spent in the border zone minus the expected border-zone area proportion of 75%. Data are presented as mean ± SD with individual animals overlaid; *n* = 19 young degus (10 males and 9 females) and 31 old degus (13 males and 18 females). Statistical significance was determined using two-tailed Mann–Whitney U tests.

We first assessed age-associated locomotor activity and center-border exploration during a 5 min open-field tracking window. Quantitative analysis showed that old degus moved a significantly greater total distance than young degus (*p* = 0.0004; Figure 2B). Old degus also exhibited higher mean velocity (*p* = 0.0009; Figure 2C), increased moving frequency (*p* = 0.0002; Figure 2D), and longer cumulative movement duration (*p* = 0.0003; Figure 2E).

We next quantified center-zone duration and area-corrected thigmotaxis score to assess center-border exploration. Old degus showed increased center-zone duration (*p* = 0.0003; Figure 2F) and a reduced area-corrected thigmotaxis score (*p* = 0.0004; Figure 2G). Together, these findings indicate that aged degus displayed increased locomotor activity together with reduced thigmotaxis and increased center-zone exploration during open-field exposure.

### 3.2. Aged Degus Show Increased Locomotor Activity in a Chamber-Based Behavioral Apparatus

**Figure 3:**
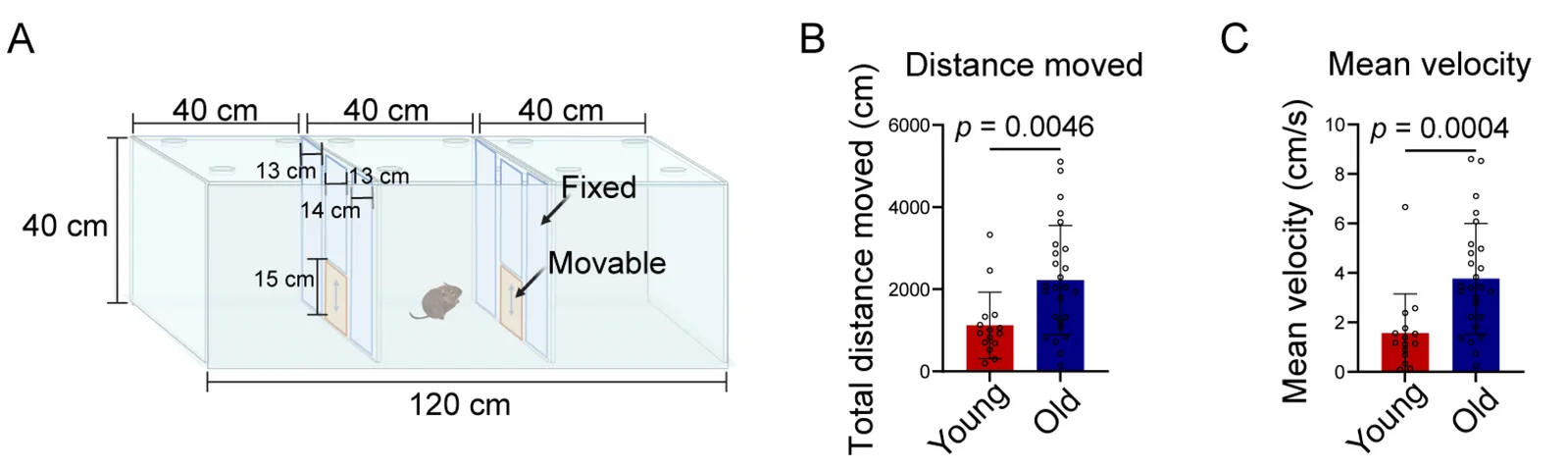
Chamber-based locomotor activity is increased in aged degus. (**A**) Schematic of the chamber-based behavioral apparatus. (**B**,**C**) Quantification of total distance moved (**B**) and mean velocity (**C**) during the 10 min chamber-based tracking window. Data are presented as mean ± SD with individual animals overlaid; young, *n* = 15 (7 males and 8 females); old, *n* = 26 (10 males and 16 females). Statistical significance was determined using two-tailed Mann–Whitney U tests.

We next asked whether the age-associated increase in locomotor activity observed in the open-field arena was also present in a structured chamber-based behavioral environment. Movement was analyzed during a 10 min chamber-based tracking window (Figure 3A). Quantitative analysis showed that old degus moved a significantly greater total distance than young degus (*p* = 0.0046; Figure 3B) and showed higher mean velocity during chamber-based testing (*p* = 0.0004; Figure 3C). Together, these findings indicate that increased locomotor activity in aged degus was not limited to the open-field arena but was also detected in a structured chamber-based behavioral environment.

### 3.3. Aged Degus Show Reduced Social Novelty Discrimination

**Figure 4:**
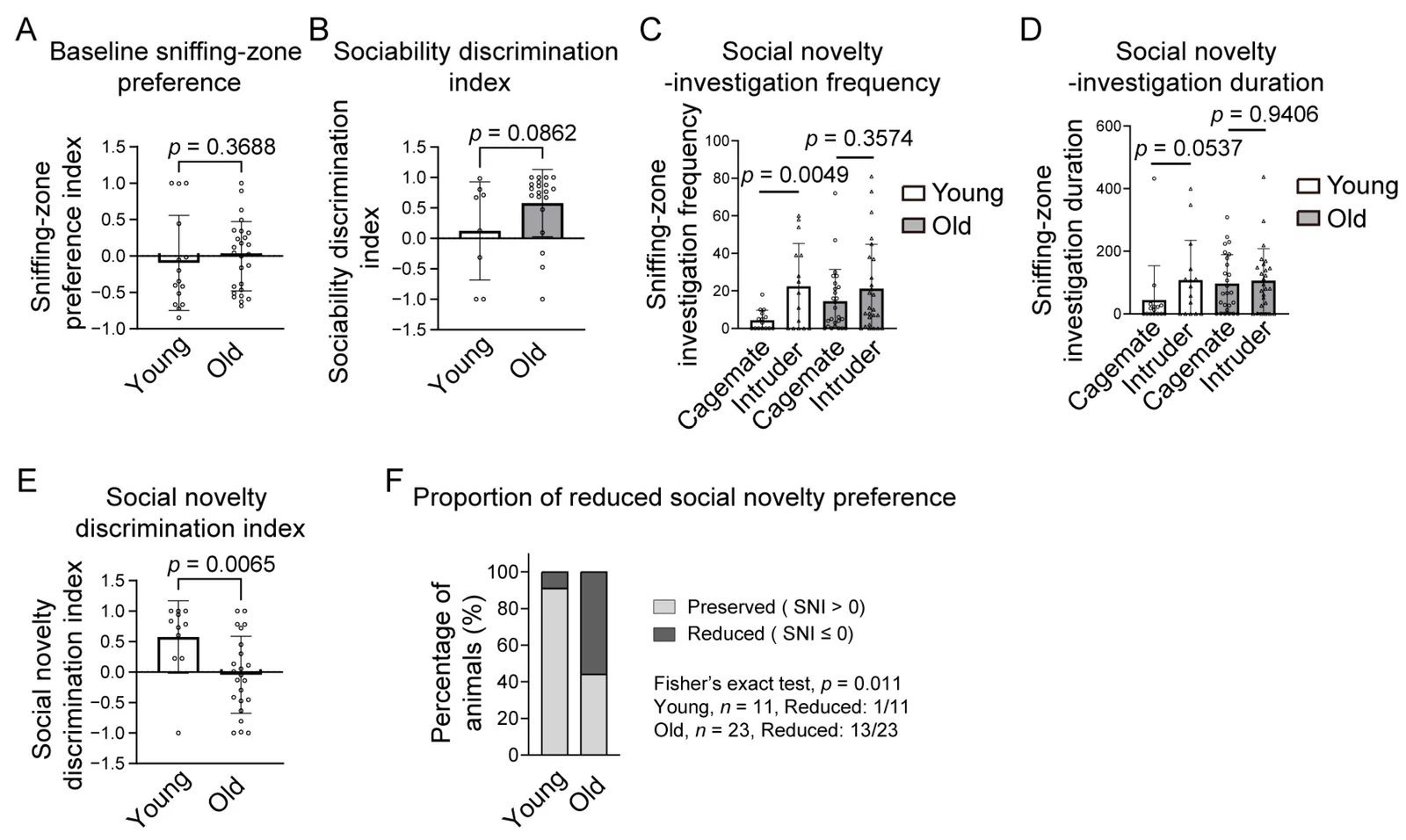
Aged degus show reduced social novelty discrimination. (**A**) Quantification of baseline sniffing-zone preference during the habituation phase. The sniffing-zone preference index was calculated using cumulative sniffing-zone duration in the two enclosure-associated sniffing zones before introduction of social stimuli. (**B**) Quantification of sociability during the sociability phase. The sociability discrimination index was calculated as (cagemate − empty enclosure)/(cagemate + empty enclosure) using cumulative sniffing-zone duration. (**C**) Quantification of sniffing-zone entry frequency toward the familiar cagemate and novel intruder during the social novelty phase. Circles represent the familiar cagemate, and triangles represent the novel intruder. (**D**) Quantification of cumulative sniffing-zone duration toward the familiar cagemate and novel intruder during the social novelty phase. (**E**) Quantification of the social novelty discrimination index calculated as (novel intruder − familiar cagemate)/(novel intruder + familiar cagemate) using cumulative sniffing-zone duration. (**F**) Proportion of animals with reduced social novelty discrimination, defined as a social novelty discrimination index ≤ 0. For social novelty phase frequency and duration analyses, young, *n* = 15 and old, *n* = 26. For social novelty discrimination index and proportion analyses, young, *n* = 11 and old, *n* = 23 after exclusion of animals with undefined index values. Data are presented as mean ± SD with individual animals overlaid where applicable. Paired comparisons between familiar cagemate and novel intruder sniffing-zone entry frequency or cumulative sniffing-zone duration were performed using two-tailed Wilcoxon matched-pairs signed-rank test. Young and old index values were compared using two-tailed Mann–Whitney U tests. Proportions were compared using Fisher’s exact test.

We next assessed social behavior using a three-chamber social behavior assay consisting of habituation, sociability, and social novelty phases. To determine whether age groups differed in baseline occupancy of the enclosure-associated sniffing zones before the introduction of social stimuli, we first quantified the sniffing-zone discrimination index during the habituation phase. Young and old degus did not differ significantly in baseline sniffing-zone preference index (*p* = 0.3688; Figure 4A), suggesting that age-associated differences observed in later phases were unlikely to be explained by pre-existing side bias.

We then assessed sociability during the sociability phase. The sociability discrimination index did not differ significantly between young and old degus (*p* = 0.0862; Figure 4B), indicating that aged animals did not show a significant loss of general social approach under these testing conditions.

During the social novelty phase, young degus showed significantly higher sniffing-zone entry frequency toward the novel intruder than toward the familiar cagemate (*p* = 0.0049; Figure 4C). In contrast, old degus did not show a significant difference in sniffing-zone entry frequency between the novel intruder and familiar cagemate (*p* = 0.3574; Figure 4C). When cumulative sniffing-zone duration was analyzed, neither young nor old degus showed a significant within-group difference between the novel intruder and familiar cagemate zones (young, *p* = 0.0537; old, *p* = 0.9406; Figure 4D). However, the duration-based social novelty discrimination index was significantly lower in old degus than in young degus (*p* = 0.0065; Figure 4E). Using a social novelty discrimination index ≤ 0 to define reduced social novelty discrimination, 13 of 23 old degus (56.5%) met this criterion, compared with 1 of 11 young degus (9.1%; Fisher’s exact test, *p* = 0.011; Figure 4F). Together, these findings indicate that aging in laboratory-housed degus is associated with reduced novelty-directed social investigation and impaired social novelty discrimination, whereas baseline sniffing-zone preference and sociability were not significantly altered between age groups.

### 3.4. Sex-Stratified Analysis of Open-Field and Social Novelty Behavioral Metrics

**Figure 5:**
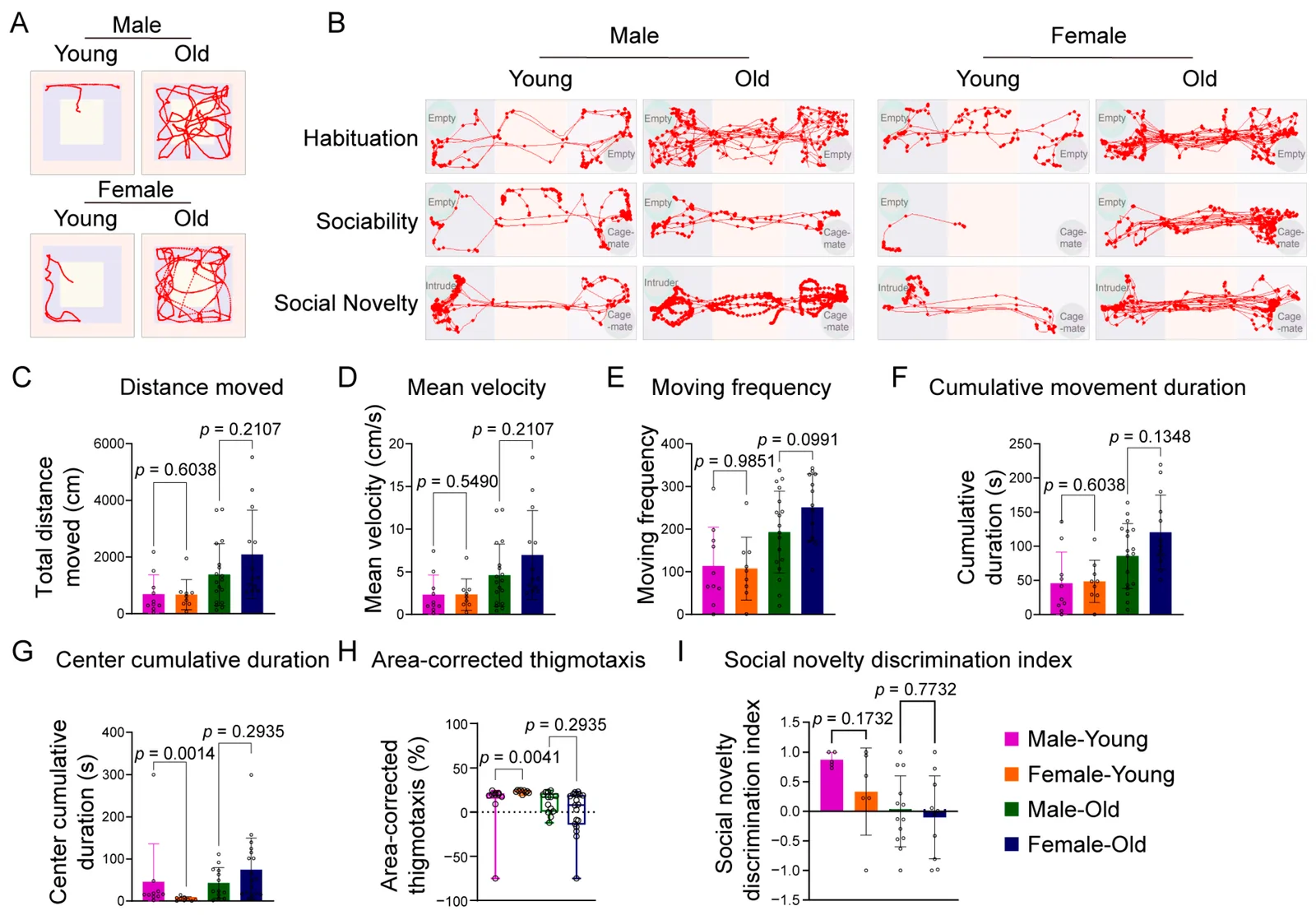
Sex-stratified analysis of open-field and social novelty behavioral metrics within young and old degus. (**A**) Representative open-field locomotor trajectories from young and old male and female degus during the 5 min open-field tracking window. (**B**) Representative three-chamber locomotor trajectories from young and old male and female degus during the habituation, sociability, and social novelty phases. These representative panels are provided for visualization of tracking patterns; quantitative analyses are shown in the corresponding panels. (**C**–**H**) Sex-stratified quantification of open-field behavioral metrics, including total distance moved (**C**), mean velocity (**D**), moving frequency (**E**), cumulative movement duration (**F**), center-zone cumulative duration (**G**), and area-corrected thigmotaxis score (**H**). Open-field analysis included young males, *n* = 10; young females, *n* = 9; old males, *n* = 13; and old females, *n* = 18. (**I**) Sex-stratified quantification of the social novelty discrimination index. Social novelty analysis included young males, *n* = 5; young females, *n* = 6; old males, *n* = 10; and old females, *n* = 13. Data are presented as mean ± SD with individual animals overlaid. Within each age group, males and females were compared using two-tailed Mann–Whitney U tests. *p* values are shown.

Because both male and female animals were included in the behavioral cohort, we performed exploratory sex-stratified analyses to examine male–female differences within each age group. Example tracking patterns from male and female animals are shown for open-field and three-chamber testing (Figure 5A,B). In the young cohort, males and females did not differ significantly in total distance moved, mean velocity, moving frequency, or cumulative movement duration (Figure 5C–F). However, young males showed a significantly longer center-zone cumulative duration than young females (*p* = 0.0014; Figure 5G) and a significantly lower area-corrected thigmotaxis score (*p* = 0.0041; Figure 5H), indicating a sex-associated difference in center–border exploration. In the old cohort, no significant male–female differences were detected in any of the open-field locomotor or center–border exploration measures examined (Figure 5C–H). The social novelty discrimination index also did not differ significantly between males and females within either the young (*p* = 0.1732) or old (*p* = 0.7732) cohort (Figure 5I). Thus, sex-associated differences were selectively detected in center–border exploration in young degus but were not consistently observed across other open-field or social novelty measures.

### 3.5. Manual Ethological Scoring Reveals Increased Rearing and Fecal Boli in Aged Degus

**Figure 6:**
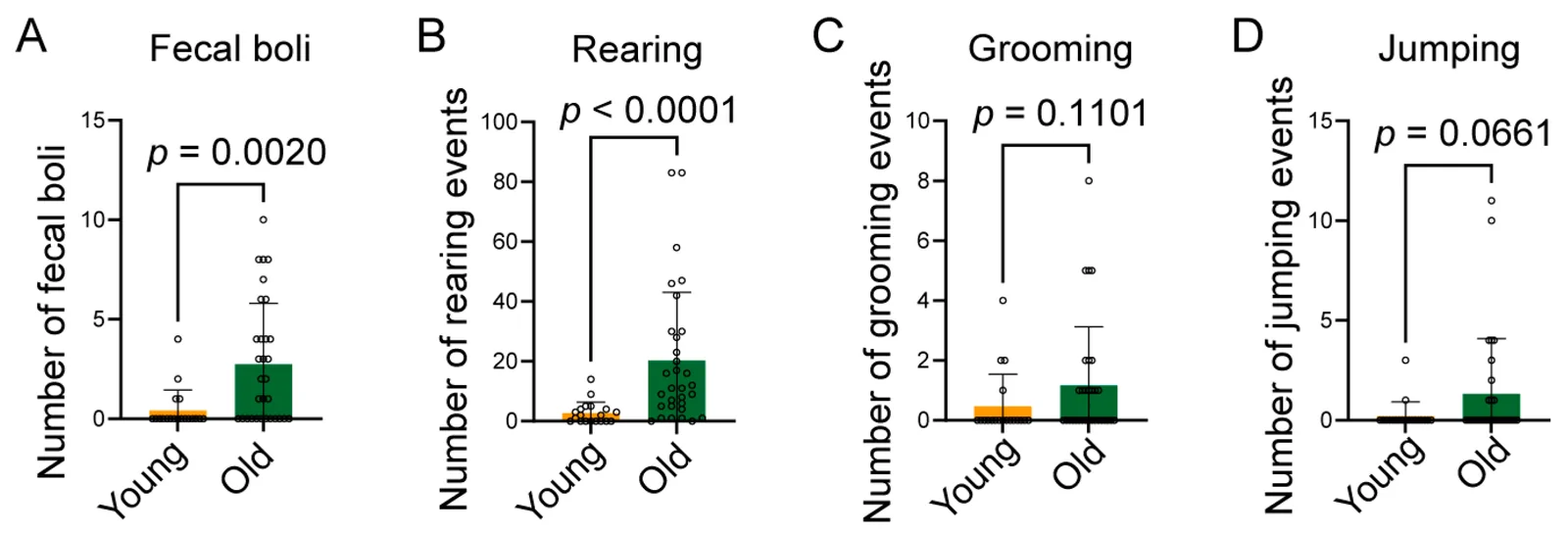
Manual ethological scoring of open-field behaviors in young and old degus. (**A**–**D**) Quantification of manually scored open-field behaviors during the 5 min open-field exposure, including fecal boli (**A**), rearing events (**B**), grooming events (**C**), and jumping events (**D**). Old degus showed significantly increased fecal boli and rearing events compared with young degus. Grooming did not differ significantly between age groups, whereas jumping showed a non-significant trend toward increase in old animals. Data are presented as mean ± SD with individual animals overlaid; young, *n* = 19 (10 males and 9 females); old, *n* = 31 (13 males and 18 females). Statistical significance was determined using two-tailed Mann–Whitney U tests.

To complement automated behavioral tracking, we manually scored selected ethological behaviors during a 5 min open-field exposure. This analysis captured behavioral features that are not fully represented by distance- or velocity-based tracking metrics, including fecal boli, rearing, grooming, and jumping. Old degus produced significantly more fecal boli than young degus (*p* = 0.0020; Figure 6A) and showed a marked increase in rearing events (*p* < 0.0001; Figure 6B). Grooming events did not differ significantly between age groups (*p* = 0.1101; Figure 6C), whereas jumping events showed a non-significant trend toward increase in old animals (*p* = 0.0661; Figure 6D). Together, these manually scored behaviors indicate that aged degus exhibit increased fecal output and vertical exploration during open-field exposure, supporting an age-associated shift in stress-related and exploratory behavioral responses beyond automated locomotor measures.

## 4. Discussion

This study reveals age-associated behavioral changes across locomotor, exploratory, stress-related, and social novelty domains in laboratory-housed *Octodon degus*. Rather than showing a generalized reduction in behavioral engagement, aged degus displayed a mixed phenotype characterized by increased locomotor activity during open-field and chamber-based testing, increased rearing and fecal output, reduced thigmotaxis, and reduced social novelty discrimination. Together, these findings indicate that aging in degus is associated with coordinated changes in exploratory activation, stress-related responses, center avoidance, and novelty-directed social investigation.

A prominent finding was increased locomotor activity in aged degus during behavioral testing. Aged animals traveled farther, moved faster, initiated more movement bouts, and spent more time moving in both the open-field and three-chamber apparatuses. Because reduced locomotion or insufficient exploration can confound the interpretation of social and cognitive assays [18,19], the reduced social novelty discrimination observed here is unlikely to be explained simply by general behavioral inactivity.

This pattern contrasts with findings in conventional laboratory rodents, in which aging commonly reduces locomotion and impairs sensorimotor performance. Altun et al. reported gradual declines in open-field locomotion and rearing in rats, with accelerated impairments in balance and gait in late life [20]. Likewise, Shoji and Miyakawa observed age-dependent reductions in locomotor activity across several behavioral assays in C57BL/6J mice [21], consistent with other mouse and rat studies [22,23,24,25]. However, the present study measured activity only within behavioral testing arenas, not in the home cage, and did not directly assess strength, gait, balance, or coordination. The findings therefore indicate greater behavioral activation during testing rather than increased overall daily activity or improved motor function.

The open-field phenotype was also not consistent with a simple unidirectional change in anxiety-like behavior. Aged animals spent more time in the center zone and showed reduced thigmotaxis, but these measures can also be influenced by locomotor activity, exploratory drive, novelty response, and risk assessment [26,27,28,29]. Reduced thigmotaxis occurred alongside increased locomotion and rearing, whereas fecal boli, an index of emotionality- or stress-related autonomic output, were also increased. Together, the combination of increased exploratory activity and greater fecal boli production is consistent with altered arousal and stress responsiveness to a novel environment rather than simply reduced anxiety. Age-related changes in novelty response or habituation remain possibilities requiring direct investigation.

The reduction in social novelty discrimination provides a more direct link to cognitive and social-recognition-related behavioral domains. Degus are highly social animals, and successful performance in the three-chamber social novelty test requires animals to distinguish a novel conspecific from a familiar one and to direct investigation toward the novel social stimulus. Young degus showed greater investigation of the novel intruder than the familiar cagemate, whereas old degus did not show a significant novelty-directed investigation pattern. Consistently, aged degus had a lower social novelty discrimination index and a larger proportion of animals with reduced social novelty discrimination. Because baseline sniffing-zone preference and sociability index were not significantly different between age groups, this phenotype is less likely to reflect pre-existing zone bias or a general loss of social approach. Instead, it may reflect reduced social novelty discrimination, altered social recognition memory, reduced attention to social cues, changes in novelty-directed social motivation, or age-related sensory changes that affect detection of social olfactory cues.

These behavioral changes may be relevant to neurodegenerative aging because AD and related dementias can involve affective, arousal-related, stress-related, and social disturbances in addition to memory impairment [30,31,32,33,34,35,36,37,38]. Behavioral phenotypes in AD mouse models are heterogeneous, ranging from hypoactivity to hyperactivity and showing variable anxiety-like and social behaviors depending on genotype, age, sex, genetic background, disease stage, and testing paradigm [18,39,40,41,42,43,44,45,46,47,48,49,50,51]. Previous degu studies have reported age-associated impairments in burrowing, recognition memory, social memory, and maze performance, sometimes together with AD-like neuropathological or synaptic changes [4,5,6,10,11,12]. However, Steffen et al. and Bourdenx et al. reported no consistent spontaneous age-related AD- or PD-like brain pathology in captive degu cohorts [8,9]. Notably, the relevant aged samples were limited to 4 animals in a 7-animal quantitative cohort in Steffen et al. and 3 animals older than 4 years in the 15-animal cohort examined by Bourdenx et al. Such limited representation of advanced age may have been insufficient to detect heterogeneous or relatively infrequent AD-like neuropathological changes, even if these changes were present in a subset of animals. These limitations highlight the importance of incorporating comprehensive behavioral phenotyping before tissue collection in future studies, thereby allowing behaviorally impaired and behaviorally preserved subgroups to be identified and compared neuropathologically.

Several limitations should be considered. This study was designed to define age-associated behavioral phenotypes rather than identify causal mechanisms. Locomotor activity was not measured in the home cage, and center-zone exploration and thigmotaxis do not directly measure anxiety state. Social novelty behavior may reflect multiple processes, including social recognition, olfaction, attention, motivation, and exploratory drive. Although the animals passed routine veterinary assessment, olfactory function was not directly tested; therefore, age-related sensory changes cannot be excluded. In the sociability phase, the empty enclosure did not contain a nonsocial object matched for physical complexity, although this limitation did not apply to the social novelty phase, in which both enclosures contained social stimuli. Animals with zero cumulative duration in both sniffing zones were excluded from index-based analyses because the discrimination index was undefined. Testing day was not fully balanced across age and sex groups; therefore, potential testing-day effects cannot be excluded, particularly when interpreting the sex-associated center–border differences in young animals. Finally, neuropathological, endocrine, and molecular measurements were not obtained from the same animals. Future studies correlating these behavioral measures with amyloid, tau, neuroinflammation, corticosterone, and other aging-related markers will be required to determine their underlying biological significance.

The coordinated alterations observed across exploratory, stress-related, and social novelty domains suggest that aged degus may exhibit broader changes in behavioral regulation that are relevant to brain aging and neurodegenerative vulnerability. Future studies linking these behavioral measures with amyloid pathology, tau phosphorylation, neuroinflammation, corticosterone signaling, and age-associated transcriptional or metabolic alterations will help determine whether they reflect neurodegenerative vulnerability, broader changes in arousal and stress responsiveness, or both. Such analyses will be important for establishing how behavioral heterogeneity in naturally aging degus relates to biological mechanisms of brain aging and to phenotypes relevant to age-related neurodegenerative disease.

## 5. Conclusions

In conclusion, this study defines an age-associated behavioral profile in laboratory-housed *Octodon degus* across locomotor, exploratory, stress-related, and social novelty domains. Aged degus did not show a generalized reduction in behavioral engagement; instead, they displayed increased locomotor activity during open-field and chamber-based testing, increased rearing and fecal output, reduced thigmotaxis, and a lower social novelty discrimination index. This pattern suggests that aging in degus is associated with enhanced exploratory activation, altered stress-related autonomic responses, reduced center avoidance, and reduced novelty-directed social investigation. Sex-associated differences were limited to greater center-zone exploration in young males, with no significant differences in the other behavioral measures. Together, these findings provide a functional behavioral framework for future studies linking degu aging phenotypes to neurodegenerative vulnerability, behavioral heterogeneity, and molecular or neuropathological features of brain aging.

## Data Availability

The data presented in this study are available within the article. Additional data supporting the findings of this study are available from the corresponding author upon reasonable request.
